# Small RNA deep sequencing revealed microRNAs’ involvement in modulating cellular senescence and immortalization state

**DOI:** 10.1016/j.psj.2022.102474

**Published:** 2023-01-04

**Authors:** Chen Zhu, Lei Zhang, Mohammad Heidari, Shuhong Sun, Shuang Chang, Qingmei Xie, Yongxing Ai, Kunzhe Dong, Huanmin Zhang

**Affiliations:** ⁎ USDA, Agricultural Research Service, Avian Disease and Oncology Laboratory, East Lansing, MI 48823, USA; † Michigan State University, East Lansing, MI 48824, USA; ‡ Institute of Special Wild Economic Animal and Plant Science, Chinese Academy of Agricultural Sciences, Changchun, Jilin 130112, China; § College of Animal Science and Veterinary Medicine, Shandong Agricultural University, Tai'an, Shandong 271018, China; # College of Animal Science, South China Agricultural University, Guangzhou, Guangdong 510642, China; ǁǁ College of Animal Science, Jilin University, Changchun, Jilin 130062, China; ¶ Department of Pharmacology and Toxicology, Augusta University, Augusta, GA 30912, USA

**Keywords:** spontaneously immortalized cells, microRNAs, microRNA expressions, predicted target genes, pathways

## Abstract

Unlike rodent cells, spontaneous immortalization of avian cells and human cells is a very rare event. According to patent publications and current literature, there are no more than 4 spontaneously immortalized chicken embryo fibroblast (**CEF**) cell lines established up to date. One of those cell lines is ADOL (Avian Disease and Oncology Laboratory) ZS-1 cell line, which was established by continuous passaging of the CEFs derived from the specific pathogen free (**SPF**) 0.TVB*S1 (commonly known as rapid feathering susceptible or RFS) genetic line of chickens. The RFS genetic line of chickens was developed and has been maintained on the SPF chicken farm of USDA-ARS facility, ADOL, in East Lansing, Michigan, which is known as one of a few lines of chickens that are free of any known avian endogenous virus genes. To explore potential roles that epigenetic factors may play in modulating cellular senescence processes and spontaneous immortalization state, total RNAs extracted from samples of the RFS primary CEFs, RFS CEFs reached the 21st passage, and the ZS-1 cells were subjected to small RNA sequencing. Collectively, a total of 531 miRNAs was identified in the 3 types of samples. In contrast to the primary CEF samples, 50 miRNAs were identified with significantly differential expression only in the 21st passage samples; a different subset of 63 differentially expressed miRNAs was identified only in the ZS-1 samples; the majority of differentially expressed miRNAs identified in both the 21st passage CEF and the ZS-1 samples were more or less directionally consistent. Gene Ontology analysis results suggested that the epigenetic factor, miRNAs, plays a role in modulating the cellular senescence and spontaneous immortalization processes through various bioprocesses and key pathways including ErbB and MAPK signaling pathways. These findings provided the experimental and bioinformatic evidence for a better understanding on the epigenetic factor of miRNAs in association with cellular senescence and spontaneous immortalization process in avian cells.

## INTRODUCTION

Cell lines play critical roles in biomedical research and in prevention, diagnosis, and even therapeutic treatment of diseases, including infectious diseases, of human and animals (Bennani et al., 2020; Buonerba et al., 2020; Zhu et al., 2020; Yin et al., 2021). Of all the cell lines developed through varied means, spontaneously immortalized and endogenous retrovirus-free (EV free) avian cell lines are unique and truly rare resources standing out by their unique properties, including, but not limited to, close resemblance to the cellular characteristics from which they were derived. Spontaneously immortalized and endogenous retrovirus-free avian cell line enables the most relevant models for in vitro study of the target cells since those cells were not subjected to biological nor chemical treatment during the process of immortalization and are free of endogenous retroviruses. Cells of such types are also critical and ideal for biomanufacturing vital bioreagents, such as antibodies and vaccines (Garber et al., 2009; Giotis et al., 2019; Lee et al., 2020, 2021; Lin et al., 2020; Chungu et al., 2021; He et al., 2021).

There are only a few spontaneously immortalized chicken cell lines reported (Kool, 2019). The very first non-vial, non-viral protein, and non-chemically transformed avian immortalized cell line is known as the DF-1 cell line (Foster and Foster, 1997), which was derived by continuously passaging of chicken embryo fibroblastic (**CEF**) cells of the line-0 chickens, a EV free line of specific pathogen free (**SPF**) chickens that has been developed and maintained on the experimental farm of the USDA-ARS, Avian Disease and Oncology Laboratory (**ADOL**) at East Lansing, Michigan, since 1985 (Bacon et al., 2000).

The DF-1 line of cells has been widely used as substrates in virus propagation, recombinant protein expression, recombinant virus production, verification of gene expression and gene overexpression, modeling of gene functions and functions of foreign genes, and exploring host-pathogen interactions. DF-1 cells have also been used in CRISPR/Cas9 gene editing studies (Zhao et al., 2017; Sid and Schusser, 2018). In particular to study of host-pathogen interactions between avian cells and avian leukosis viruses (**ALV**), it was confirmed that the spontaneously immortalized DF-1 cells are more suitable than regular primary CEF (Maas et al., 2006).

Another EV free SPF line of chickens has been developed and maintained on the USDA-ARS, ADOL farm (Zhang et al., 2008). This genetic line of chickens was derived from a F_2_ flock that was initiated from a cross between the line-0 and another ADOL line of chickens known as 0.44-VB*S1*-EV21* (Bacon, Hunt and Cheng, 2000). This new addition of the SPF line of chickens was named as 0.TVB*S1 (commonly known as rapid feathering susceptible or RFS). The *TVB* refers to the tumor virus B locus of chicken genome, a gene that encodes the cellular receptors necessary to mediate successful infection of the subgroup B, D, and E ALV. There are 3 major alleles at this locus, TVB*S1, TVB*S3, and TVB*R, that have been commonly detected and described in chicken (Klucking et al., 2002; Zhang et al., 2007). Briefly, TVB*S1 encodes receptors capable of mediating subgroup B, D, and E ALV infection. This is the most susceptible *TVB* allele. TVB*S3 encodes receptors that permit subgroup B D ALV infection but block subgroup E ALV from entering an infection cycle (Adkins et al., 2001). TVB*R encodes a dysfunctional receptor that is incapable of mediating any B, D, or E subgroup ALV infection (Klucking, Adkins and Young, 2002). The line-0 is TVB*S3/S*3 homozygous, susceptible to subgroup B and D ALV but resistant to subgroup E ALV infection (C/E). The new EV free SPF line, 0.TVB*S1, is TVB*S1/S*1 homozygous, susceptible to all subgroup B, D, and E ALV infection (C/0) (Zhang, Bacon and Fadly, 2008).

Use of the primary CEF of the 0.TVB*S1 line of chickens, a spontaneously immortalized chicken cell line, ADOL ZS-1, was developed (Zhang, 2016). The primary CEFs were dissociated and grown in culture with continuous passage until senescence began obviously observable. The cells were then concentrated during the cell senescence phase to maintain a 30% to 69% culture confluence. Eventually, the remained subpopulation of cells continued proliferation over 48 passages in culture. The ADOL ZS-1 cell line is free of EV and susceptible to all subgroups of ALV, including the subgroup E. The cells of the ADOL ZS-1 cell line and its subclones thereof may be used for inter alia the production of viral agents, including recombinant viral agents, expression or overexpression of recombinant proteins, exploring or validating gene or foreign gene functions, host-virus interactions, and diagnostic assays. The ADOL ZS-1 cell line is available at ATCC (https://www.atcc.org/products/pta-121623).

The epigenetic factor, miRNAs, is reportedly a class of key modulators of cellular senescence, and cellular senescence is a key player in tumor suppression processes (Liu et al., 2012; Abdelmohsen and Gorospe, 2015). This study aimed to explore miRNA expression of the ADOL ZS-1 cell line, and to provide bioinformatic evidence to elucidate the epigenetic factor's roles that may be involved with cellular senescence and/or the immortalization process free of all known transformation agents.

## MATERIALS AND METHODS

### Cell Samples and Total RNA Preparation

Three types of cell samples were used in this study, which were primary CEFs, 21st passage CEFs, and the ADOL ZS-1 line of cells. Primary CEFs were recovered from fertilized eggs of the 0.TVB*S1 line by aseptically removing the embryonic torso of the 10-day-old embryos, mincing the tissue, dissociating the cells in a trypsin-containing Gibco Dulbecco's Modified Eagle Medium (**DMEM**), and collecting and growing the dissociated cells in enriched DMEM culture medium. After incubation, cells of this primary culture were recovered by trypsinization. Three independent preparations of the primary culture were sampled for total RNA extractions. The cells of the primary culture were also plated in fresh culture medium containing 6% fetal calf serum for additional passages. Between the 10th and 25th passages, a working solution of Trypsin (0.25% Trypsin in PBS) was used to briefly loosen and to discard some of the senescent (aging) cells from the surface of the petri dish before completely dissociating the cells in the petri dish by trypsinization to passage the cells. Three random plates of cells of the 21st passage were sampled for total RNA extractions. After repeated passage, spontaneously immortalized cells were obtained from the mixed cell population in a pure form. These cells were recovered and designated ZS-1. Cells of the ZS-1 cell line are immortal and have been passaged over 40 passages. Three plates of cells of the ADOL ZS-1 line were sampled for total RNA extractions. The total RNA samples of the cell samples from the primary culture, the 21st passage, and the 48th passage ADOL ZS-1 cell line, which all shared the same genetic lineage of the 0.TVB*S1 line of chickens, were extracted with TRIzol reagent (Invitrogen, Carlsbad, CA) following the manufacturer's instructions.

### Small RNA Sequencing

Small RNA libraries were prepared using the Illumina Small RNA Library Preparation Kit (Illumina, San Diego, CA, Cat. 63600). The completed libraries were quantified, and quality controlled (**QC**) using a combination of Qubit dsDNA High Sensitivity (**HS**), Caliper LabChipGX HS DNA and Kapa Illumina Library qPCR Quantification assays (Science, 2012; Mehta et al., 2018; Bronkhorst et al., 2020). The small RNA libraries were then sequenced on an Illumina HiSeq 2500 platform in a single-end 50 base format. All these procedures were carried out at and by the Genomics group of the Research Technology Support Facility on Michigan State University campus (Genomics - Research Technology Support Facility (msu.edu)).

### Small RNA Sequence Reads Analysis for miRNAs and Target Gene Prediction

The small RNA_Seq reads that passed QC were analyzed following the similar procedures described earlier (Zhang et al., 2020a). Briefly, the pass-filter (**PF**) reads (the number of clusters that passed Illumina's “Chastity filter”) were analyzed with miRDeep* software package (v3.8) using the default parameters (An et al., 2013) except the adapter sequence and chicken genome-specific index files. The adapter sequence used in this analysis was TGG AAT TCT CGG GTG CCA AGG AAC TCC AGT CAC (Illumina); and the chicken genome build index (build_bwt_idx) files were constructed based on the chromosome information of the galGal 5.0 genome assembly along with the latest version of “gga.gff3” file downloaded from the miRbase website (Kozomara et al., 2019).

Target genes of the differentially expressed miRNAs were predicted using the built-in target gene prediction function in miRDeep*, which employees the most commonly used target gene prediction tool, TargetScan, to predict target genes of known and novel miRNAs (Lewis et al., 2005).

### Droplet Digital PCR Analysis to Validate the RNA Sequence Data

A small random sample of identified miRNAs was subjected to Droplet Digital PCR (**ddPCR**) analysis to validate the levels of small RNA_Seq reads. Primers were customarily designed for ddPCR analysis of each of the sampled miRNAs using mature sequences following the procedures described by Balcells et al. (2011). The cDNA samples used in ddPCR validation were reversely transcribed from individual total RNA samples using the iScript RT Supermix Kit (Cat No. 170-8841) and following the manufacturer's instruction (Bio-Rad Laboratories, Inc., Hercules, CA). A ddPCR reaction of 25 *μ*L in final volume was initially prepared per miRNA per biological sample containing 2 *μ*L of cDNA (60 *n*g/*μ*L), 12.5 *μ*L of EvaGreen Supermix (final concentration: 1X; Cat No. 1864034), 0.5 *μ*L of each forward and reverse primers (200 nM; synthesized by Eurofins Genomics, Huntsville, AL), and 9.5 *μ*L of nuclease-free water. Of which, 20 *μ*L were loaded into one of 8 sample channels of a DG8 cartridge (Cat No. 1864008, Bio-Rad). Each oil well was loaded with 70 *μ*L of droplet generating oil (Cat No. 1864006, Bio-Rad). The loaded DG8 cartridges were placed on a QX200 droplet generator (Bio-Rad) to generate the digital droplets. Forty *μ*L of the generated droplet emulsion per sample were transferred to a well in a 96-well PCR plate followed by polymerase chain reaction with EvaGreen on a C1000 Thermal Cycler (Bio-Rad). The cycling conditions were 95°C for 5 min, followed by 40 cycles of 95°C for 15 s, 58°C for 60 s, and a final extension step of 90°C for 5 min. The droplets post PCR were read well by well on a QX200 droplet reader (Bio-Rad). PCR-positive and PCR-negative droplets in each of the wells were counted and analyzed with the QuantaSoft Software (Version 1.7, Bio-Rad). The primers designed for ddPCR validation of the selected miRNAs are given in Table 2.

### Differentially Expressed miRNA Identification and GO Terms Enrichment Analysis

The number of reads per microRNA for each biological sample was counted using HTSeq (Anders et al., 2015). In each of the pairwise comparisons (between the primary and the 21st passage CEF; between the primary and the ZS-1 line of cells), differentially expressed microRNAs were identified by use of a custom R script encompassing the DESeq R package (2.1.0). A filter criterion of *FDR* < 0.05 and FC > 2 was enforced. For some of the differentially expressed miRNAs that ended up with a zero-statistic estimate for a normalized average TPM (baseMeanA or baseMeanB) in a contrast, an arbitrary small value of 5 was assigned to substitute the zero in order to computer a numeric fold change, and then a log_2_ fold change value for easier comprehension of the estimates. To better understand the functional involvements of the identified microRNAs differentially expressed between the primary and the ZS-1 line of cells, the predicted target gene lists of the upregulated microRNAs and the downloaded miRNAs that were identified only in the ZS-1 cell samples in contrast to the primary CEF samples were subjected to GO Terms and pathway analysis independently using the g:Proflier2 (https://biit.cs.ut.ee/gprofiler/gost) online tools with the following options: Organism: Gallus gallus; Statistical domain scope: All known genes; Significance threshold: Bonferroni correction; User threshold: 0.01 (Reimand et al., 2007).

## RESULTS AND DISCUSSION

### Small RNA Sequence Reads of the Total RNA Samples

Small RNA sequencing generated an average of 49.98 million PF reads per type of the primary, 21st passage, and 48th passage (ZS-1 cell line) cell samples, with a range of 13.9 to 20.9 and 45.9 to 54.9 million reads per biological sample and per type of cell-samples, respectively (Table 1). The raw sequence datasets (accession numbers: SAMN11676622 - SAMN11676630) are available at the Sequence Read Archive (**SRA**) under the National Center for Biotechnology Information (NCBI) website with an assigned BioProject SRA accession: PRJNA543529 (https://www.ncbi.nlm.nih.gov/sra/PRJNA543529).

**Table 1:** A summary of small RNA sequence reads and statistics.

Sample	Sample no.	Pass-filter (PF) reads	PF reads ≥ Phred quality score 30
Primary CEF	1	16,247,160	96.7%
	2	20,853,108	96.8%
	3	17,821,315	96.7%
Subtotal		54,921,583	
21st passage CEF	1	14,178,472	96.6%
	2	15,936,586	96.7%
	3	15,805,086	96.7%
Subtotal		45,920,144	
ZS-1 line of cells	1	17,850,533	97.0%
	2	17,328,707	96.9%
	3	13,922,326	96.9%
Subtotal		49,101,566	
Grand total		149,943,293	

### Validation of the Small RNA_Seq Expression Data by ddPCR

**Figure 1:**
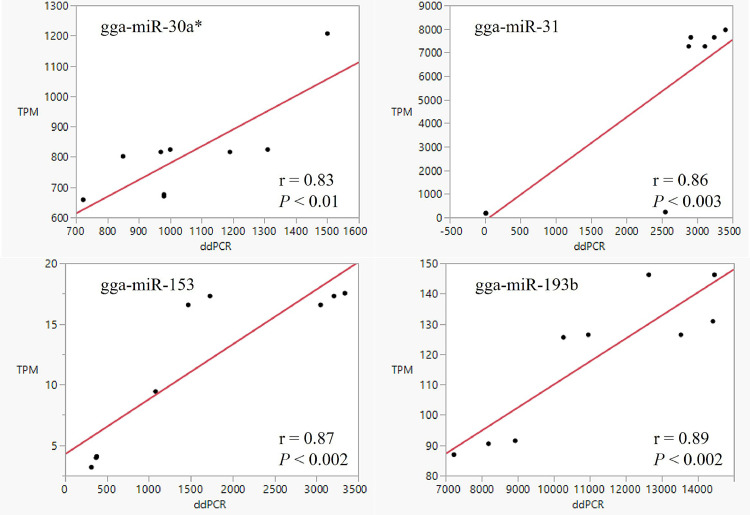
A bivariate plot illustrating the moderately high associations between the droplet digital PCR generated absolute quantifications (ddPCR) and the normalized small RNA sequence data (TPM) of a set of randomly selected miRNAs. This result supports the validity of the small RNA sequence data generated in this study.

A randomly selected small set of miRNAs was subjected to ddPCR analysis using customer designed primers (Table 2). The absolute quantification of the randomly selected four miRNAs in the same sets of total RNA samples by ddPCR was analyzed against the corresponding normalized PF reads (TPM) of the small RNA sequence data with a bivariate model. The correlation coefficients between the two sets of expression data for the examined miRNAs ranged from r = 0.83 to r = 0.89 with a range of statistic *P* < 0.01 to *P* < 0.002 (Figure 1), which provided a highly positive support in validation of the small RNA sequence data generated in this study and the estimates of the miRNA expression derived from the small RNA sequence datasets.

**Table 2:** Primers designed and used for ddPCR validation of selected miRNA expression.

miRNA	Sequence	Forward primer (5’ 3’)	Reverse primer (5’ 3’)
gga-miR-30a	TGTAAACATCCTCGACTGGAAGC	GCGCAGTGTAAACATCCTCGA	CAGGTCCAGTTTTTTTTTTTTTTTCCAGT
gga-miR-31	AGGCAAGATGTTGGCATAGCTG	GCAGAGGCAAGATGTTGGCAT	CAGGTCCAGTTTTTTTTTTTTTTTCAGCTA
gga-miR-153	TTGCATAGTCACAAAAGTGATC	GCGCAGTTGCATAGTCACAAAAG	CCAGGTCCAGTTTTTTTTTTTTTTTGATCA
gga-miR-193b	AACTGGCCCACAAAGTCCCGCT	CGCAGAACTGGCCCACAAAG	GTCCAGTTTTTTTTTTTTTTTAGCGGGACT

### MicroRNA Profiles of the Primary CEFs, 21st Passage CEFs, and the ADOL ZS-1 Line of Cells

A total of 531 miRNAs was identified at least in 3 of the nine bio-samples of the primary, 21st CEFs, and the ZS-1 line of cells. Two hundred and twenty-nine of those were known miRNAs and the rest of 302 were novel miRNAs (Supplementary Table S1). Of which, 504, 459, and 422 miRNAs were identified in the primary, 21st passage, and ZS-1 cell samples, respectively (Supplementary Tables S2–S4). A set of 50 miRNAs was reportedly identified in the DF-1 cells by small RNA sequencing (Chen et al., 2019). Thirty-one of those were overlapped with miRNAs identified in this study (Supplementary Table S4). However, those 31 miRNAs were not only identified in the ZS-1 samples, but also in the primary and the 21st passage CEF samples. Another study reported that gga-miR-375 was identified in the DF-1 cells (Zhang et al., 2020b), which was identified in the primary CEF and the ZS-1 samples (Supplementary Table S4), but not in the 21st passage CEF samples. By comparing the 3 lists of identified miRNAs of the 3 types of cell samples, a total of 42 miRNAs (18 known and 24 novel miRNAs) was found present only on the list of the primary CEF samples; 5 novel miRNAs were identified only in the 21st passage CEF samples; and a set of different 5 novel miRNAs was detected only in the ZS-1 cells. The identification of different unique subsets of miRNAs in each of the 3 types of cell samples indicated that miRNAs and miRNA expression may have been involved with cell passaging and even were associated with cellular senescence and immortalization processes. Furthermore, there were 62, 25, and 17 miRNAs identified in both the primary and 21st CEFs, the primary CEFs and ZS-1 cells, and the 21st passage CEFs and the ZS-1 samples, respectively (Supplementary Table S5). It was noticeable that there were over 5 times of specific miRNAs detected only in the primary CEF samples in contrast to both the 21st passage CEF and the ZS-1 cell samples. There were 5 miRNAs detected either only in the 21st passage CEF samples or the ZS-1 cell samples, and all were novel miRNAs. Based on the reported observations during the development of the very first spontaneously immortalized chicken embryo fibroblastic cell line, the DF-1, and subsequent studies for characteristics of the DF-1 cells, normal primary CEFs entered a highly senescent phase post 10 passages with a slow growth rate in tissue culture; by the 40th passage, a survived homogeneous population of cells grew rapidly (Himly et al., 1998; Lassiter et al., 2014). Therefore, it is speculated that the unique subsets of miRNAs that detected only in the primary CEFs, 21st passage CEFs, and the ZS-1 cells may be more or less associated with the maintenance of normal cell growth, reduced normal cell growth and cell senescence, and spontaneous transformation in addition to other factors and other miRNAs that were detected in more than one type of cell samples but with differential expressions between the different types of cell samples.

### Differentially Expressed miRNAs of the ADOL ZS-1 Cells and the 21st Passage Chicken Embryo Fibroblasts in Contrast to the Primary Chicken Embryo Fibroblasts

A total of 188 (96 known and 92 novel) miRNAs was identified with significantly differential expression (2.30E-194 ≤ *P* ≤ 2.10E-02 and 6.06E-192 ≤ *FDR* ≤ 4.50E-02) between the ADOL ZS-1 line of cells and the primary CEFs. Fifty-five of those miRNAs were upregulated and the rest of 133 were downregulated in expression in the ADOL ZS-1 line of cells (Table 3). There were 175 (100 known and 75 novel) miRNAs identified with significantly differential expression (3.59E-268 ≤ *P* ≤ 2.05E-02 and 9.45E-266 ≤ *FDR* ≤ 4.95E-02) between the 21st passage and the primary CEFs. Sixty-two of those miRNAs were upregulated and the rest of 113 miRNAs were downregulated when the CEFs reached the 21st passage (Table 4). With a close look at the 2 lists of differentially expressed miRNAs derived from the 2 contrasts, between the ZS-1 cells and the primary CEFs and between the 21st passage and the primary CEFs, eighteen miRNAs were upregulated and 45 miRNAs were downregulated in expression only in the ZS-1 cells; 24 miRNAs were upregulated and 26 miRNAs were downregulated in expression only in the 21st passage CEFs; the rest of differentially expressed miRNAs were all directionally consistent more or less upregulated or downregulated in expression between the 2 contrasts (Supplementary Table S6). Increasing evidence indicates that miRNAs play vital roles in a variety of biological processes including maintaining cellular mechanism and proliferation (Yin et al., 2021). The miR-21 is reported as an oncogene and plays a key role in resisting programmed cell death in cancer and targets apoptosis (Buscaglia and Li, 2011) The gga-mir-21 was identified in all 3 types of samples in this study, and it was overexpressed significantly in both the 21st passage CEF samples and the ZS-1 samples in contrast to the primary CEF samples (Supplementary Table S6), suggesting that gga-mir-21 may have exerted influence in overcoming cellular senescence in the 21st passage CEFs and in maintaining the immortalization status of the ZS-1 cells. Other differentially expressed miRNAs, including novel microRNAs, identified in the contrast between the ZS-1 samples and the primary CEF samples might play role(s) similar to the gga-mir-21, which remain to be further investigated in the future.

**Table 3:** Differentially expressed miRNAs between the ADOL ZS-1 line of cells and the primary chicken embryo fibroblasts.

miRNA	Sequence	Log_2_ FC	*P* value	*FDR*	miRNA	Sequence	Log_2_ FC	*P* value	*FDR*
gga-mir-6542	acgggacagtgctgaagactac	7.38	4.27E-31	4.78E-30	gga-mir-206	tggaatgtaaggaagtgtgtgg	−10.57	2.68E-179	3.52E-177
gga-mir-460b	tcctcattgtacatgctgtgt	3.88	2.05E-47	3.60E-46	gga-mir-206*	tggaatgtaaggaagtgtgtgg	−10.57	2.68E-179	3.52E-177
gga-mir-12221-3p	ttcgtgtgccatcgtcctttgt	3.37	1.48E-02	3.27E-02	novelMiR_332	agccactgactaacgcacattg	12.56	9.08E-03	2.14E-02
gga-mir-140	accacagggtagaaccacggac	2.69	8.78E-104	4.62E-102	novelMiR_308_2	tcccgtccccgtcggtcgcgg	8.98	5.09E-06	1.98E-05
gga-mir-148b-3p	tcagtgcatcacagaacttggt	2.63	1.66E-90	5.44E-89	novelMiR_325	ggtggacaggggtcactttt	8.46	9.36E-06	3.57E-05
gga-mir-146b	tgagaactgaattccataggcgt	2.57	1.56E-10	8.13E-10	novelMiR_343	gtgcattgtagttgcattgca	7.59	8.79E-36	1.22E-34
gga-mir-15a	tagcagcacataatggtttgt	2.50	3.58E-81	1.11E-79	novelMiR_428	ttgcatagtcacaaaagtgatcg	6.83	7.96E-22	6.35E-21
gga-mir-23b	atcacattgccagggattaccac	2.42	6.93E-64	1.58E-62	novelMiR_357	acagaatgtctctttgaaaacc	5.45	5.58E-09	2.67E-08
gga-mir-455	tatgtgcccttggactacatcg	2.30	2.27E-75	6.30E-74	novelMiR_363	ggggcgcgggggcggggg	5.30	3.79E-08	1.63E-07
gga-mir-27b	ttcacagtggctaagttctgc	2.13	2.44E-69	6.41E-68	novelMiR_350	ccggccctatcgaagctgcgcct	4.98	1.12E-06	4.45E-06
gga-mir-365-1	taatgcccctaaaaatccttat	2.06	3.81E-39	5.57E-38	novelMiR_424	aggggaggctttgctgtcgtcc	4.78	7.80E-06	3.02E-05
gga-mir-365-2	taatgcccctaaaaatccttat	2.06	3.81E-39	5.57E-38	novelMiR_403_1	tgccgactcgtctgtccgcc	3.83	6.73E-03	1.65E-02
gga-mir-21	tagcttatcagactgatgttgac	2.06	6.36E-65	1.52E-63	novelMiR_403_2	tgccgactcgtctgtccgcc	3.83	6.73E-03	1.65E-02
gga-mir-21*	tagcttatcagactgatgttgac	2.06	6.36E-65	1.52E-63	novelMiR_411	attccagtgattgatgtttggct	3.72	4.26E-03	1.08E-02
gga-mir-24	tggctcagttcagcaggaaca	1.88	1.21E-20	9.12E-20	novelMiR_412	aatgcaaagcagctgtaaaact	3.56	7.53E-03	1.83E-02
gga-mir-16-1	tagcagcacgtaaatattggtg	1.87	1.89E-26	1.85E-25	novelMiR_417	tcttacactgcagggctgagt	3.48	9.51E-03	2.21E-02
gga-mir-16-2	tagcagcacgtaaatattggtg	1.87	1.89E-26	1.85E-25	novelMiR_64	cgtgaggctgcacggggctccgt	3.35	2.36E-21	1.83E-20
gga-mir-29b-1	tagcaccatttgaaatcagtgtt	1.86	7.25E-05	2.48E-04	novelMiR_327	aaggagtccccaggctgtgctg	3.21	2.10E-02	4.50E-02
gga-mir-29b-2	tagcaccatttgaaatcagtgtt	1.86	7.25E-05	2.48E-04	novelMiR_36	aactggcctacaaagtcccagt	2.27	1.43E-33	1.68E-32
gga-mir-6561	tctttccaggcaggagctccct	1.80	6.04E-03	1.50E-02	novelMiR_101_1	ggggatgtagctcagcggtaga	1.71	2.48E-13	1.38E-12
gga-mir-29c	tagcaccatttgaaatcggtta	1.76	1.08E-25	9.81E-25	novelMiR_101_2	ggggatgtagctcagcggtaga	1.71	2.48E-13	1.38E-12
gga-mir-29a	tagcaccatttgaaatcggtta	1.76	1.13E-25	1.00E-24	novelMiR_101_3	ggggatgtagctcagcggtaga	1.71	2.48E-13	1.38E-12
gga-mir-22	aagctgccagttgaagaactgt	1.69	2.48E-45	4.20E-44	novelMiR_192	gcagggcgaggagctgccccggt	1.67	1.14E-03	3.18E-03
gga-mir-1559	ttcgatgcttgtatgctactcc	1.64	1.12E-34	1.41E-33	novelMiR_130	tctggctgccgcctactgacacc	1.67	5.02E-06	1.97E-05
gga-mir-146c	tgagaactgaattccatggactg	1.45	1.81E-07	7.49E-07	novelMiR_199	ccaggagaagcagactgtagtt	1.63	4.94E-03	1.25E-02
gga-mir-10b	taccctgtagaaccgaatttgt	1.43	2.65E-29	2.90E-28	novelMiR_46	ttggtggctgcatgctctcagc	1.56	1.34E-03	3.73E-03
gga-mir-458a	atagctctttgaatggtactgc	1.24	9.00E-10	4.51E-09	novelMiR_94	ctttttgcggtctgggcttgc	−1.04	1.26E-08	5.70E-08
gga-mir-148a	tcagtgcactacagaactttgt	1.18	2.23E-23	1.83E-22	novelMiR_18_4	agagaatcatagaatggcctgg	−1.33	5.14E-04	1.57E-03
gga-mir-12258-5p	tgcacggctgcagcctcctcacc	1.10	1.28E-08	5.77E-08	novelMiR_18_1	agagaatcatagaatggcctgg	−1.38	3.75E-04	1.16E-03
gga-mir-181a-1	aacattcaacgctgtcggtgagt	1.08	2.85E-20	2.08E-19	novelMiR_18_2	agagaatcatagaatggcctgg	−1.38	3.75E-04	1.16E-03
gga-mir-181a-2	aacattcaacgctgtcggtgagt	1.08	2.85E-20	2.08E-19	novelMiR_18_3	agagaatcatagaatggcctgg	−1.38	3.75E-04	1.16E-03
gga-mir-10c-5p	taccctgtagactcgaatttgt	−1.33	4.16E-12	2.28E-11	novelMiR_184	aattgcacggtatccatctgt	−1.46	5.98E-33	6.84E-32
gga-mir-107	agcagcattgtacagggctatc	−1.34	3.68E-26	3.46E-25	novelMiR_157	gcggccgcgggcgcggcg	−1.52	1.44E-02	3.20E-02
gga-mir-1416	tccttaactcatgccgctgtg	−1.38	7.22E-07	2.95E-06	novelMiR_82	taggtagtttcatgttgttggg	−1.54	4.80E-34	5.87E-33
gga-mir-1805	tgtattggaacactacagctcc	−1.39	9.71E-04	2.75E-03	novelMiR_173	tgatatgtttgatattaggttg	−1.91	1.59E-02	3.48E-02
gga-mir-18b	taaggtgcatctagtgcagt	−1.42	2.84E-05	1.01E-04	novelMiR_127_1	tctttggttatctagctgtatga	−2.37	1.12E-19	7.67E-19
gga-mir-187	tcgtgtcttgtgttgcagcca	−1.53	3.92E-03	1.01E-02	novelMiR_127_2	tctttggttatctagctgtatga	−2.37	1.12E-19	7.67E-19
gga-mir-204-1	ttccctttgtcatcctatgcct	−1.54	1.04E-14	6.41E-14	novelMiR_180	cccgcggccgtcgcacagcgct	−2.81	6.24E-03	1.55E-02
gga-mir-204-2	ttccctttgtcatcctatgcct	−1.54	1.04E-14	6.41E-14	novelMiR_95	cgcccccctcgggcgggc	−2.84	3.76E-04	1.16E-03
gga-mir-211	ttccctttgtcatcctatgcct	−1.54	1.04E-14	6.41E-14	novelMiR_49	cggagcgaggagggctcggaga	−2.93	1.22E-02	2.77E-02
gga-mir-196-2	taggtagtttcatgttgttggg	−1.57	1.03E-34	1.33E-33	novelMiR_22	tggtgagaagcgtgctgtggagc	−2.96	1.03E-02	2.37E-02
gga-mir-196-1	taggtagtttcatgttgttggg	−1.58	7.28E-35	9.57E-34	novelMiR_149	accgtggctttagattgttact	−3.13	4.67E-44	7.68E-43
gga-mir-106	aaaagtgcttacagtgcaggtag	−1.62	4.04E-24	3.42E-23	novelMiR_196_1	ggcggcggcggcggccgcgggc	-3.24	1.28E-24	1.10E-23
gga-mir-20b	caaagtgctcatagtgcaggtag	−1.71	1.21E-27	1.27E-26	novelMiR_196_2	ggcggcggcggcggccgcgggc	−3.28	1.70E-27	1.76E-26
gga-mir-126	cattattacttttggtacgcg	−2.02	8.35E-14	4.99E-13	novelMiR_103_1	gcgggcggcgcggcggcg	−3.44	9.52E-03	2.21E-02
gga-mir-1684a	aagtatgaggaaatggagatct	−2.06	4.37E-11	2.37E-10	novelMiR_103_2	gcgggcggcgcggcggcg	−3.44	1.74E-04	5.74E-04
gga-mir-146a	tgagaactgaattccatgggttg	−2.27	1.40E-14	8.55E-14	novelMiR_103_3	gcgggcggcgcggcggcg	−3.44	9.52E-03	2.21E-02
gga-mir-9-2	tctttggttatctagctgtatga	−2.37	1.12E-19	7.67E-19	novelMiR_103_4	gcgggcggcgcggcggcg	−3.44	9.52E-03	2.21E-02
gga-mir-9-1	tctttggttatctagctgtatga	−2.37	1.08E-19	7.67E-19	novelMiR_151	gggccggggaccgccgcc	−3.44	2.07E-02	4.45E-02
gga-mir-9-1*	tctttggttatctagctgtatga	−2.37	1.08E-19	7.67E-19	novelMiR_26_7	ggcggcgcggcggcggcg	−3.44	1.38E-02	3.10E-02
gga-mir-1397	tgcattgcgacgggttatatc	−2.71	7.65E-04	2.23E-03	novelMiR_206	gcctacggccatcccaccc	−3.45	1.81E-16	1.16E-15
gga-mir-1397*	tgcattgcgacgggttatatc	−2.71	7.65E-04	2.23E-03	novelMiR_26_2	ggcggcgcggcggcggcg	−3.45	1.30E-02	2.91E-02
gga-mir-6615	ttggggacaccatcagaacagcc	−2.84	8.73E-09	4.07E-08	novelMiR_26_6	ggcggcgcggcggcggcg	−3.45	1.30E-02	2.91E-02
gga-mir-1779	agacgtggactggaacacctgag	−2.85	5.63E-03	1.42E-02	novelMiR_39	gagactggcaggactggcagt	−3.53	1.51E-02	3.31E-02
gga-mir-1329	acagtgatcacgttacgatggat	−2.94	8.34E-09	3.95E-08	novelMiR_171	cggggcgcgggggcgggg	−3.62	2.56E-03	6.88E-03
gga-mir-551	gcgacccatacttggtttcag	−3.12	9.73E-11	5.17E-10	novelMiR_28_2	gggcggcggcggcggccg	−3.67	9.56E-03	2.21E-02
gga-mir-12209-3p	tcgtgaccttctctttgtatt	−3.53	1.59E-02	3.48E-02	novelMiR_21_2	gccggggcggggcgggcc	−3.71	9.01E-03	2.14E-02
gga-mir-212-3p	taacagtctacagtcatggct	−3.67	5.00E-28	5.37E-27	novelMiR_11_3	gcggcggcggcggggggg	−3.72	8.48E-03	2.02E-02
gga-mir-6606	gaggagcgggaggagcggga	−3.68	1.04E-02	2.38E-02	novelMiR_11_5	gcggcggcggcggggggg	−3.72	8.48E-03	2.02E-02
gga-mir-7-2	tggaagactagtgattttgttgt	−3.73	8.07E-03	1.95E-02	novelMiR_11_7	gcggcggcggcggggggg	−3.72	8.48E-03	2.02E-02
gga-mir-7-3	tggaagactagtgattttgttgt	−3.79	6.43E-03	1.59E-02	novelMiR_191	tgcacctcgcgctgcgggc	−3.80	6.00E-03	1.50E-02
gga-mir-133a-1	tttggtccccttcaaccagctgt	−4.25	2.30E-194	6.06E-192	novelMiR_238	gcgcgccgggcccggggc	−3.89	2.05E-02	4.44E-02
gga-mir-133a-2	tttggtccccttcaaccagctgt	−4.25	2.30E-194	6.06E-192	novelMiR_163_2	gcaggcgggcgggcgggc	−3.90	4.05E-03	1.04E-02
gga-mir-184	tggacggagaactgataagggt	−4.27	2.13E-23	1.78E-22	novelMiR_26_1	ggcggcgcggcggcggcg	−3.99	2.48E-04	7.95E-04
gga-mir-1744	acttcaacaggagcaagactga	−4.46	2.45E-04	7.95E-04	novelMiR_26_3	ggcggcgcggcggcggcg	−3.99	2.48E-04	7.95E-04
gga-mir-1703	cagaggctgtaggtcccgtgct	−4.50	2.10E-04	6.91E-04	novelMiR_26_4	ggcggcgcggcggcggcg	−3.99	1.55E-07	6.48E-07
gga-mir-1724	tgctgagcgttggctgcgctgc	−4.54	2.22E-05	7.99E-05	novelMiR_26_5	ggcggcgcggcggcggcg	−3.99	2.48E-04	7.95E-04
gga-mir-1677	ttgacttcagtaggagcaggatt	−4.57	2.54E-35	3.42E-34	novelMiR_31	ggcgcagcgttggattttt	−4.01	2.64E-03	7.04E-03
gga-mir-1b	tggaatgttaagaagtatgtat	−4.75	3.90E-05	1.37E-04	novelMiR_32_2	tgaggcccgatgtgtcattcctg	−4.06	2.14E-03	5.79E-03
gga-mir-489	tgacatcatatgtacggctgct	−4.87	9.51E-165	1.00E-162	novelMiR_32_3	tgaggcccgatgtgtcattcctg	−4.06	2.14E-03	5.79E-03
gga-mir-1a-1	tggaatgtaaagaagtatgtat	−5.10	4.51E-49	8.17E-48	novelMiR_28_1	gggcggcggcggcggccg	−4.13	1.53E-03	4.23E-03
gga-mir-1a-2	tggaatgtaaagaagtatgtat	−5.10	4.51E-49	8.17E-48	novelMiR_174	gcccgccggcccccggcg	−4.15	1.44E-03	3.98E-03
gga-mir-133c	tttggtccccttcaaccagctg	−5.30	1.54E-141	1.16E-139	novelMiR_71	ggcgcggcggcggcggggg	−4.22	9.78E-04	2.75E-03
gga-mir-2188	aaggtccaacctcacatgtcct	−5.45	5.95E-08	2.50E-07	novelMiR_161	cgggccggggaccgccgcc	−4.26	4.59E-22	3.72E-21
gga-mir-137	ttattgcttaagaatacgcgtag	−5.57	1.37E-08	6.09E-08	novelMiR_169	tcaatgagagcacggtctgcc	−4.27	7.73E-04	2.25E-03
gga-mir-218-1	ttgtgcttgatctaaccatgtg	−5.59	2.96E-93	1.04E-91	novelMiR_98	cggcgcgccgggcccgggg	−4.61	1.05E-04	3.53E-04
gga-mir-218-2	ttgtgcttgatctaaccatgtg	−5.59	2.96E-93	1.04E-91	novelMiR_194	gcggcggcggccgcgggc	−4.94	9.06E-06	3.48E-05
gga-mir-202	ttcctatgcatatacttcttt	−5.72	1.79E-09	8.90E-09	novelMiR_188	cggcgcgcggctcggcgcggc	−5.18	1.07E-06	4.31E-06
gga-mir-1720	aagcaacgagaggtcggtctga	−5.79	1.20E-13	6.86E-13	novelMiR_106	cgaaaatgaccggggtggacct	−5.21	7.44E-07	3.01E-06
gga-mir-10a	taccctgtagatccgaatttgt	−5.93	6.03E-132	3.97E-130	novelMiR_91	atctggctgcgacatctgtcacc	−5.27	6.37E-07	2.62E-06
gga-mir-429	taatactgtctggtaatgccg	−6.04	4.14E-57	8.71E-56	novelMiR_27	ccgccggcggcgccgggc	−5.53	2.22E-08	9.81E-08
gga-mir-122-1	tggagtgtgacaatggtgtttg	−6.30	1.04E-13	6.05E-13	novelMiR_236	cgtgaggctgcacggggctccg	−5.63	7.16E-04	2.12E-03
gga-mir-142	cccataaagtagaaagcactac	−6.39	2.09E-21	1.64E-20	novelMiR_209	tggagtgtgacaatggtgtttg	−6.30	1.04E-13	6.05E-13
gga-mir-375	tttgttcgttcggctcgcgtt	−6.85	2.43E-55	4.91E-54	novelMiR_19	ggggcgcgggggcgggggg	−6.36	3.02E-14	1.82E-13
gga-mir-499	ttaagacttgtagtgatgttt	−6.99	1.06E-33	1.27E-32	novelMiR_73	cgcttgatcttgattttcagta	−6.97	3.55E-21	2.71E-20
gga-mir-205a	tccttcattccaccggagtctg	−7.01	2.62E-163	2.30E-161	novelMiR_175	actgatagaagctgagacct	−7.29	4.51E-26	4.16E-25
gga-mir-1729*	ctactcggtgagtaaggatagc	−7.25	2.30E-25	2.01E-24	novelMiR_102	ttgcatagtcacaaaagtgatc	−7.29	3.34E-26	3.20E-25
gga-mir-200b	taatactgcctggtaatgatgat	−7.31	1.84E-26	1.85E-25	novelMiR_203	gcattggtggttcagtgg	−7.65	6.09E-05	2.11E-04
gga-mir-200a	taacactgtctggtaacgatgtt	−7.94	1.73E-39	2.68E-38	novelMiR_104	ttctggtgatgagacctttgtcc	−7.79	5.46E-36	7.76E-35
gga-mir-205b	cccttcattccaccggaatctg	−8.28	2.77E-78	8.10E-77	novelMiR_141	agccggggatgatttctgcct	−8.11	5.35E-44	8.53E-43
gga-mir-490	caacctggaggactccatgctgt	−8.67	1.79E-62	3.93E-61	novelMiR_8_1	ctgtcacgcgggagaccggggt	−8.14	7.90E-19	5.26E-18
gga-mir-1662	ttgacatcatcatacttgggat	−9.53	2.25E-103	1.08E-101	novelMiR_8_2	ctgtcacgcgggagaccggggt	−8.14	7.90E-19	5.26E-18
gga-mir-203a	gtgaaatgtttaggaccacttg	−9.76	9.65E-118	5.64E-116	novelMiR_193	tttccaaccttcgtgattctga	−8.34	7.60E-51	1.48E-49
gga-mir-133b	tttggtccccttcaaccagct	−9.99	4.11E-99	1.66E-97	novelMiR_146	ctcggatcggccccggcggggt	-11.86	3.85E-100	1.69E-98

**Table 4:** Differentially expressed miRNAs between the 21st passage and the primary chicken embryo fibroblasts.

miRNA	Sequence	Log_2_ FC	*P* value	*FDR*	miRNA	Sequence	Log_2_ FC	*P* value	*FDR*
gga-mir-6542	acgggacagtgctgaagactac	7.41	1.15E-30	1.37E-29	gga-mir-200b	taatactgcctggtaatgatgat	−7.34	1.71E-26	1.73E-25
gga-mir-31	aggcaagatgttggcatagctg	5.36	1.67E-256	2.92E-254	gga-mir-499	ttaagacttgtagtgatgttt	−7.49	1.44E-43	1.85E-42
gga-mir-458a	atagctctttgaatggtactgc	4.24	1.24E-142	5.03E-141	gga-mir-200a	taacactgtctggtaacgatgtt	−7.96	2.57E-39	3.21E-38
gga-mir-29b-1	tagcaccatttgaaatcagtgtt	3.18	1.54E-19	1.23E-18	gga-mir-203a	gtgaaatgtttaggaccacttg	−8.49	3.18E-105	9.28E-104
gga-mir-29b-2	tagcaccatttgaaatcagtgtt	3.18	1.54E-19	1.23E-18	gga-mir-490	caacctggaggactccatgctgt	−8.70	1.44E-61	2.52E-60
gga-mir-29c	tagcaccatttgaaatcggtta	2.79	8.88E-64	1.71E-62	gga-mir-375	tttgttcgttcggctcgcgtt	−8.75	1.63E-63	2.96E-62
gga-mir-29a	tagcaccatttgaaatcggtta	2.79	9.13E-64	1.71E-62	gga-mir-429	taatactgtctggtaatgccg	−9.00	1.18E-73	2.58E-72
gga-mir-460b	tcctcattgtacatgctgtgt	2.65	4.50E-14	2.78E-13	gga-mir-133b	tttggtccccttcaaccagct	−10.02	1.52E-128	4.98E-127
gga-mir-140	accacagggtagaaccacggac	2.52	9.10E-76	2.28E-74	gga-mir-489	tgacatcatatgtacggctgct	−10.14	3.59E-268	9.45E-266
gga-mir-21	tagcttatcagactgatgttgac	2.28	8.39E-64	1.70E-62	gga-mir-205b	cccttcattccaccggaatctg	−10.29	6.75E-75	1.61E-73
gga-mir-21*	tagcttatcagactgatgttgac	2.28	8.39E-64	1.70E-62	gga-mir-206	tggaatgtaaggaagtgtgtgg	−10.60	5.55E-155	3.24E-153
gga-mir-33-1	gtgcattgtagttgcattgca	2.11	1.42E-22	1.27E-21	gga-mir-206*	tggaatgtaaggaagtgtgtgg	−10.60	5.55E-155	3.24E-153
gga-mir-15a	tagcagcacataatggtttgt	2.06	1.42E-46	2.07E-45	novelMiR_332	agccactgactaacgcacattg	14.21	0.00E+00	0.00E+00
gga-mir-1663	tggcatccagaacagcggtac	1.97	3.81E-03	1.15E-02	novelMiR_308_2	tcccgtccccgtcggtcgcgg	10.08	1.65E-145	8.70E-144
gga-mir-16-1	tagcagcacgtaaatattggtg	1.96	1.15E-47	1.77E-46	novelMiR_325	ggtggacaggggtcactttt	9.90	9.71E-133	3.65E-131
gga-mir-16-2	tagcagcacgtaaatattggtg	1.96	1.15E-47	1.77E-46	novelMiR_343	gtgcattgtagttgcattgca	9.89	6.54E-132	2.29E-130
gga-mir-19a	tgtgcaaatctatgcaaaactga	1.83	2.70E-03	8.30E-03	novelMiR_345_1	agcgcggcggggccggacgtt	7.17	3.14E-26	3.12E-25
gga-mir-365-1	taatgcccctaaaaatccttat	1.66	1.20E-21	1.02E-20	novelMiR_360	gcgaggggcggcggcggcggcc	6.69	2.53E-19	1.98E-18
gga-mir-365-2	taatgcccctaaaaatccttat	1.66	1.20E-21	1.02E-20	novelMiR_346	gttgatgatgataccccagaat	5.75	1.73E-10	9.80E-10
gga-mir-24	tggctcagttcagcaggaaca	1.61	2.12E-28	2.18E-27	novelMiR_357	acagaatgtctctttgaaaacc	5.52	9.54E-08	4.52E-07
gga-miR-148b-3p	tcagtgcatcacagaacttggt	1.45	1.16E-09	6.23E-09	novelMiR_330	gggcggcggcggcggccgc	5.45	1.08E-08	5.50E-08
gga-mir-148a	tcagtgcactacagaactttgt	1.38	1.72E-25	1.62E-24	novelMiR_333	ccgcccccctcgggcgggc	5.21	1.78E-07	8.28E-07
gga-mir-1559	ttcgatgcttgtatgctactcc	1.31	3.83E-19	2.96E-18	novelMiR_350	ccggccctatcgaagctgcgcct	4.76	1.29E-05	5.11E-05
gga-mir-12258-5p	tgcacggctgcagcctcctcacc	1.28	1.33E-10	7.61E-10	novelMiR_359	gcgcgcggctcggcgcggc	4.32	2.58E-04	9.11E-04
gga-mir-22	aagctgccagttgaagaactgt	1.23	7.30E-21	6.00E-20	novelMiR_323	cgtaccaaaagtaataatgggc	4.17	6.03E-04	2.01E-03
gga-mir-1729	ctactcggtgagtaaggatagc	1.18	2.03E-05	7.87E-05	novelMiR_338	tgcaacagctgctggagtggt	3.34	1.77E-02	4.45E-02
gga-mir-1729*	ctactcggtgagtaaggatagc	1.18	2.03E-05	7.87E-05	novelMiR_340	tccgctgtggcctggagcc	3.30	2.04E-02	4.95E-02
gga-mir-455	tatgtgcccttggactacatcg	1.17	1.04E-17	7.30E-17	novelMiR_327	aaggagtccccaggctgtgctg	3.29	2.05E-02	4.95E-02
gga-mir-1467-1	tctcagctacatcggtgtaaatc	1.16	1.36E-02	3.60E-02	novelMiR_46	ttggtggctgcatgctctcagc	3.01	8.80E-17	5.94E-16
gga-mir-146c	tgagaactgaattccatggactg	1.09	1.90E-17	1.30E-16	novelMiR_36	aactggcctacaaagtcccagt	2.69	2.43E-74	5.55E-73
gga-mir-6648-1	tccggcattctgaacgctcct	1.03	1.36E-02	3.60E-02	novelMiR_221	aggggtatgattctcgct	2.31	7.46E-05	2.78E-04
gga-mir-128-2	tcacagtgaaccggtctcttt	−1.12	1.43E-16	9.06E-16	novelMiR_186	tgtgcaaatccatgcaaaactga	2.16	6.36E-03	1.83E-02
gga-mir-128-1	tcacagtgaaccggtctcttt	−1.12	1.09E-16	7.01E-16	novelMiR_189	ccgcgtcgagctgagccgagc	1.96	3.31E-03	1.01E-02
gga-mir-454	tagtgcaatattgcttatagggt	−1.20	3.55E-18	2.56E-17	novelMiR_101_1	ggggatgtagctcagcggtaga	1.94	1.08E-16	7.00E-16
gga-mir-107	agcagcattgtacagggctatc	−1.41	1.41E-24	1.30E-23	novelMiR_101_2	ggggatgtagctcagcggtaga	1.94	1.08E-16	7.00E-16
gga-mir-183	tatggcactggtagaattcact	−1.49	2.37E-08	1.18E-07	novelMiR_101_3	ggggatgtagctcagcggtaga	1.94	1.08E-16	7.00E-16
gga-mir-383	agatcagaaggtgattgtggct	−1.64	1.19E-05	4.74E-05	novelMiR_59	ctgagcccacctgccccctgcag	1.60	1.89E-02	4.71E-02
gga-mir-204-1	ttccctttgtcatcctatgcct	−1.86	1.29E-18	9.54E-18	novelMiR_192	gcagggcgaggagctgccccggt	1.53	3.77E-03	1.14E-02
gga-mir-204-2	ttccctttgtcatcctatgcct	−1.86	1.29E-18	9.54E-18	novelMiR_199	ccaggagaagcagactgtagtt	1.51	1.13E-02	3.04E-02
gga-mir-211	ttccctttgtcatcctatgcct	−1.86	1.29E-18	9.54E-18	novelMiR_54	ggggaattagctcaaatggtaga	1.35	5.98E-03	1.73E-02
gga-mir-10c-5p	taccctgtagactcgaatttgt	−1.99	1.64E-14	1.03E-13	novelMiR_119	ggcggggcggtcccgggc	1.32	4.14E-03	1.24E-02
gga-mir-1731	acttgactgcaggcactgctgct	−2.03	1.11E-03	3.61E-03	novelMiR_130	tctggctgccgcctactgacacc	1.02	1.68E-02	4.31E-02
gga-mir-6615	ttggggacaccatcagaacagcc	−2.17	3.15E-06	1.33E-05	novelMiR_13	cgctagctgctctgcactgact	1.01	8.21E-06	3.30E-05
gga-mir-551	gcgacccatacttggtttcag	−2.24	4.56E-07	2.05E-06	novelMiR_116	ctgactggctcagcttgtgtct	−1.18	2.45E-03	7.57E-03
gga-mir-1329	acagtgatcacgttacgatggat	−2.42	7.06E-07	3.09E-06	novelMiR_196_2	ggcggcggcggcggccgcgggc	−1.45	2.54E-08	1.25E-07
gga-mir-460a	cctgcattgtacacactgtgt	−2.85	2.33E-44	3.14E-43	novelMiR_196_1	ggcggcggcggcggccgcgggc	−1.47	6.41E-08	3.09E-07
gga-mir-1779	agacgtggactggaacacctgag	−2.88	6.74E-03	1.93E-02	novelMiR_26_4	ggcggcgcggcggcggcg	−1.54	9.40E-03	2.56E-02
gga-mir-146a	tgagaactgaattccatgggttg	−2.94	1.73E-17	1.20E-16	novelMiR_17_1	aatcacagactggtttgggttg	−1.60	1.52E-02	3.92E-02
gga-mir-1769	agtgtgaaatctgcctgaaagtc	−3.11	1.41E-02	3.70E-02	novelMiR_17_2	aatcacagactggtttgggttg	−1.60	6.84E-04	2.26E-03
gga-mir-9-2	tctttggttatctagctgtatga	−3.26	1.52E-29	1.60E-28	novelMiR_17_3	aatcacagactggtttgggttg	−1.60	1.52E-02	3.92E-02
gga-mir-9-1	tctttggttatctagctgtatga	−3.26	1.45E-29	1.60E-28	novelMiR_17_4	aatcacagactggtttgggttg	−1.60	1.52E-02	3.92E-02
gga-mir-9-1*	tctttggttatctagctgtatga	−3.26	1.45E-29	1.60E-28	novelMiR_94	ctttttgcggtctgggcttgc	−2.00	8.68E-22	7.61E-21
gga-mir-7460	cctgactgagctctgctttctc	−3.49	1.88E-02	4.71E-02	novelMiR_111	tttggcaatggtagaactcacac	−2.10	7.21E-30	8.43E-29
gga-miR-212-3p	taacagtctacagtcatggct	−3.57	3.14E-35	3.85E-34	novelMiR_149	accgtggctttagattgttact	−2.57	1.01E-46	1.52E-45
gga-mir-10a	taccctgtagatccgaatttgt	−3.62	6.65E-48	1.09E-46	novelMiR_18_4	agagaatcatagaatggcctgg	−2.68	1.55E-09	8.24E-09
gga-mir-187	tcgtgtcttgtgttgcagcca	−3.65	7.82E-08	3.74E-07	novelMiR_18_1	agagaatcatagaatggcctgg	−2.81	4.41E-10	2.41E-09
gga-mir-1724	tgctgagcgttggctgcgctgc	−3.74	1.52E-04	5.49E-04	novelMiR_18_2	agagaatcatagaatggcctgg	−2.81	4.41E-10	2.41E-09
gga-mir-7-2	tggaagactagtgattttgttgt	−3.76	7.51E-03	2.12E-02	novelMiR_18_3	agagaatcatagaatggcctgg	−2.81	4.41E-10	2.41E-09
gga-mir-34b	aggcagtgtagttagctgattgt	−3.77	7.12E-03	2.03E-02	novelMiR_127_1	tctttggttatctagctgtatga	−3.26	1.52E-29	1.60E-28
gga-mir-124a	taaggcacgcggtgaatgcc	−3.82	5.84E-03	1.71E-02	novelMiR_127_2	tctttggttatctagctgtatga	−3.26	1.52E-29	1.60E-28
gga-mir-124a-2	taaggcacgcggtgaatgcc	−3.82	5.84E-03	1.71E-02	novelMiR_151	gggccggggaccgccgcc	−3.46	1.97E-02	4.85E-02
gga-mir-7-3	tggaagactagtgattttgttgt	−3.82	5.95E-03	1.73E-02	novelMiR_115	tcgtgaccttctctttgtatt	−3.56	1.50E-02	3.92E-02
gga-mir-184	tggacggagaactgataagggt	−3.92	9.38E-18	6.67E-17	novelMiR_171	cggggcgcgggggcgggg	−3.58	2.18E-03	6.86E-03
gga-mir-6599	tgacggatcctggctccctccg	−4.05	2.36E-03	7.34E-03	novelMiR_161	cgggccggggaccgccgcc	−3.68	1.41E-18	1.03E-17
gga-mir-1744	acttcaacaggagcaagactga	−4.49	2.28E-04	8.09E-04	novelMiR_28_2	gggcggcggcggcggccg	−3.70	9.00E-03	2.47E-02
gga-mir-1703	cagaggctgtaggtcccgtgct	−4.52	1.93E-04	6.90E-04	novelMiR_11_3	gcggcggcggcggggggg	−3.74	8.04E-03	2.21E-02
gga-mir-222b	agctacatctgattactgggtca	−4.77	3.88E-05	1.48E-04	novelMiR_11_5	gcggcggcggcggggggg	−3.74	8.04E-03	2.21E-02
gga-mir-1b	tggaatgttaagaagtatgtat	−4.78	3.42E-05	1.31E-04	novelMiR_11_7	gcggcggcggcggggggg	−3.74	8.04E-03	2.21E-02
gga-mir-1720	aagcaacgagaggtcggtctga	−4.83	1.02E-11	6.04E-11	novelMiR_191	tgcacctcgcgctgcgggc	−3.83	5.55E-03	1.66E-02
gga-mir-1a-1	tggaatgtaaagaagtatgtat	−4.94	4.53E-46	6.26E-45	novelMiR_174	gcccgccggcccccggcg	−4.17	1.34E-03	4.26E-03
gga-mir-1a-2	tggaatgtaaagaagtatgtat	−4.94	4.53E-46	6.26E-45	novelMiR_71	ggcgcggcggcggcggggg	−4.25	8.87E-04	2.92E-03
gga-mir-133a-1	tttggtccccttcaaccagctgt	−5.02	2.62E-208	2.75E-206	novelMiR_212	aggcagtgtattgttagttagct	−4.42	3.98E-04	1.35E-03
gga-mir-133a-2	tttggtccccttcaaccagctgt	−5.02	2.62E-208	2.75E-206	novelMiR_98	cggcgcgccgggcccgggg	−4.63	9.62E-05	3.52E-04
gga-mir-3525	cagccattctgcgattctgtga	−5.36	2.04E-07	9.42E-07	novelMiR_194	gcggcggcggccgcgggc	−4.97	8.10E-06	3.28E-05
gga-mir-2188	aaggtccaacctcacatgtcct	−5.47	5.48E-08	2.67E-07	novelMiR_188	cggcgcgcggctcggcgcggc	−5.21	9.45E-07	4.11E-06
gga-mir-137	ttattgcttaagaatacgcgtag	−5.59	1.23E-08	6.21E-08	novelMiR_106	cgaaaatgaccggggtggacct	−5.25	6.12E-07	2.70E-06
gga-mir-1397	tgcattgcgacgggttatatc	−5.65	6.19E-09	3.19E-08	novelMiR_26_5	ggcggcgcggcggcggcg	−5.26	5.97E-07	2.66E-06
gga-mir-1397*	tgcattgcgacgggttatatc	−5.65	6.19E-09	3.19E-08	novelMiR_91	atctggctgcgacatctgtcacc	−5.31	1.18E-06	5.09E-06
gga-mir-202	ttcctatgcatatacttcttt	−5.75	1.61E-09	8.48E-09	novelMiR_27	ccgccggcggcgccgggc	−5.56	1.91E-08	9.59E-08
gga-mir-218-1	ttgtgcttgatctaaccatgtg	−5.81	6.22E-143	2.73E-141	novelMiR_95	cgcccccctcgggcgggc	−5.81	6.68E-10	3.62E-09
gga-mir-218-2	ttgtgcttgatctaaccatgtg	−5.81	6.22E-143	2.73E-141	novelMiR_209	tggagtgtgacaatggtgtttg	−6.33	8.50E-14	5.14E-13
gga-mir-133c	tttggtccccttcaaccagctg	−6.17	1.35E-168	1.01E-166	novelMiR_73	cgcttgatcttgattttcagta	−7.00	2.80E-21	2.34E-20
gga-mir-122-1	tggagtgtgacaatggtgtttg	−6.33	8.50E-14	5.14E-13	novelMiR_175	actgatagaagctgagacct	−7.32	4.52E-26	4.41E-25
gga-mir-1662	ttgacatcatcatacttgggat	−6.93	2.11E-83	5.85E-82	novelMiR_141	agccggggatgatttctgcct	−8.14	8.05E-44	1.06E-42
gga-mir-1805	tgtattggaacactacagctcc	−7.13	2.66E-23	2.41E-22	novelMiR_276	tgtgcaaatccatgcaaaactg	−10.85	4.15E-06	1.73E-05
gga-mir-205a	tccttcattccaccggagtctg	−7.14	9.39E-120	2.91E-118	novelMiR_146	ctcggatcggccccggcggggt	−11.89	1.51E-81	3.98E-80
gga-mir-1677	ttgacttcagtaggagcaggatt	−7.23	2.74E-50	4.66E-49	novelMiR_92	ctgtgcgtgtgacagcggct	−13.43	3.22E-180	2.82E-178
gga-mir-142	cccataaagtagaaagcactac	−7.31	5.09E-26	4.87E-25					

### Predicted Target Genes of the Differentially Expressed miRNAs Observed in the Contrast between the ZS-1 Cells and the Primary CEFs

A total of 11,499 target genes was predicted for the 188 differentially expressed miRNAs identified in the contrast between the ADOL ZS-1 cell samples and the primary CEFs (Supplementary Table S7). A further close look of the target gene list led to 2 short lists of target genes, one was a list of 5,436 target genes predicted for 18 upregulated miRNAs in expression, which were only identified in the ZS-1 cell samples in contrast to the primary CEF samples (Supplementary Tables S6, S8); the other one was a list of 4,757 target genes predicted for 45 downregulated miRNAs in expression, which were only identified in the ZS-1 cell samples in contrast to the primary CEF samples (Supplementary Tables S6, S9). To better understand how, if any, the miRNAs would be involved in overcoming the process of cell senescence and/or maintaining a stable spontaneous immortalization state, the latter two relatively short lists of target genes were independently subjected to GO Terms analysis to explore potential roles through which the differentially expressed miRNAs may play in modulating cellular senescence and immortalization processes.

### Target-Gene-Set Enrichment in GO Terms and Pathways

**Figure 2:**
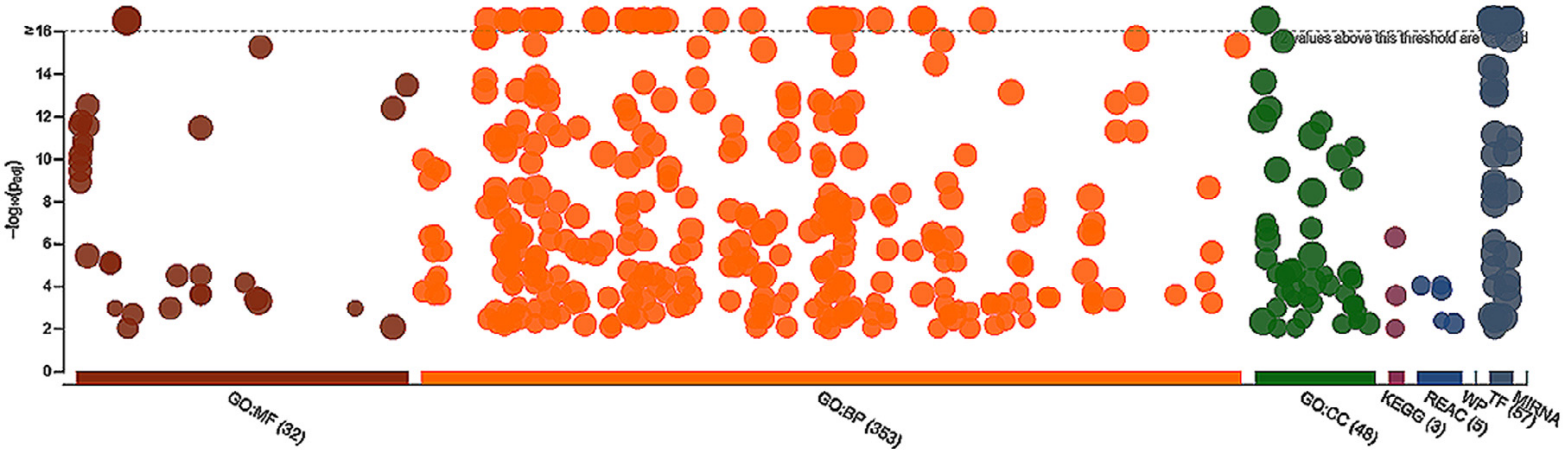
A Manhattan plot illustrating GO Terms enrichments of target genes of the upregulated miRNAs. These differentially expressed miRNAs were detected only in the ZS-1 cell samples in contrast to the primary chicken embryo fibroblast samples. The enrichment of the GO Terms MF (molecular function), BP (biological processes), CC (cellular component), and the KEGG (Kyoto Encyclopedia of Genes and Genomics) pathways, REAC (reactome) pathways, WP (WiKi) pathways, as well as TF (transcription factor) and MIRNA (microRNA target base) is depicted, respectively, in the plot, suggesting strong and wide involvements of the upregulated set of miRNAs in biological processes in the ZS-1 cells.

**Figure 3:**
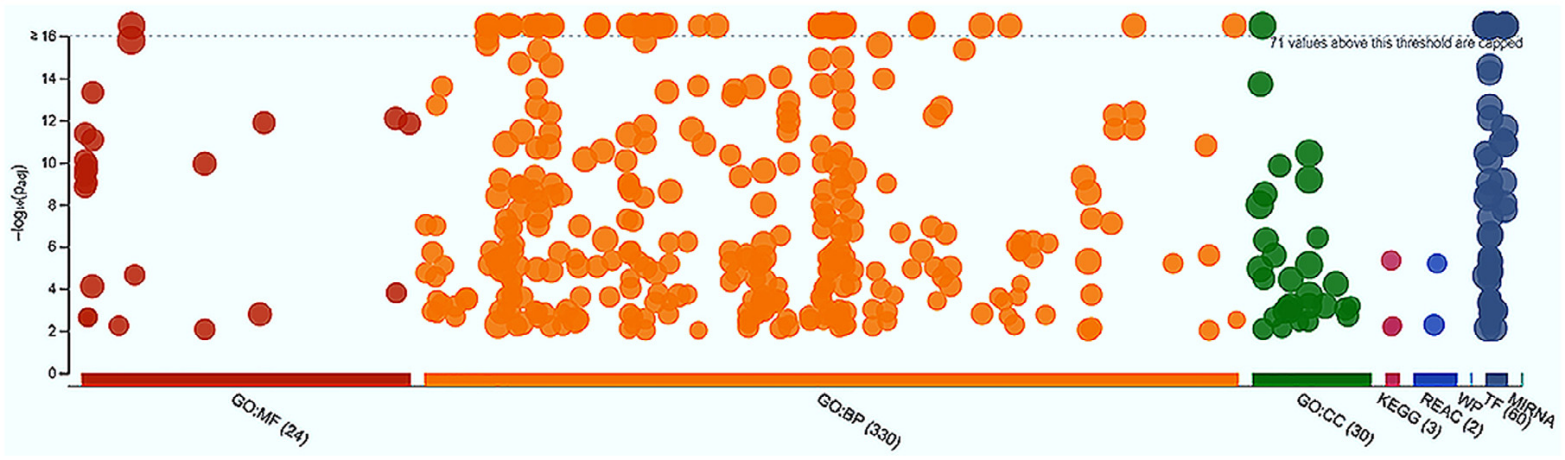
A Manhattan plot illustrating GO Terms enrichments of target genes of the downregulated miRNAs. These differentially expressed miRNAs were detected only in the ZS-1 cell samples in contrast to the primary chicken embryo fibroblast samples. The enrichments of the GO Terms MF, BP, CC, and the KEGG pathways, REAC pathways, WP pathways, as well as TF and MIRNA are depicted, respectively, in the plot, which also suggested strong and wide involvements of the downregulated set of miRNAs in biological processes through their set of target genes in the ZS-1 cells.

Two sets of target genes of the upregulated and downregulated miRNAs identified only in the ZS-1 cell samples in contrast to the primary CEF samples were independently subjected to GO Terms enrichment analyses and the outputs of the analyses showed that both sets of the target genes were highly enriched in a variety of GO Terms and pathways (Supplementary Tables S10–S11). The target genes of the upregulated miRNAs were enriched in over thirty molecular function (**MF**), 353 biological process (**BP**), and 48 cellular component (**CC**) GO Terms, in addition to KEGG and Reactome (REAC) pathways, and 57 transcription factors (Figure 2). The target genes of the downregulated miRNAs were enriched in 24 MF, 330 BP, and 30 CC GO Terms, in addition to KEGG (MAPK, ErbB, and Endocytosis) and Reactome pathways, and 60 transcription factors (Figure 3). Studies in human diploid somatic cell population doublings revealed that the cells enter a status known as terminal proliferation arrest state of senescence due to an intrinsic mechanism involving p53 and pRB/p16^INK4^ mediated pathways. Telomere shortening is reportedly a major factor attributing to the proliferation arrest state of senescence. No GO Terms nor pathways (Supplementary Tables S10–S11) identified for the target gene lists of the upregulated and downregulated miRNAs in the ZS-1 cell samples in contrast to the primary CEF samples were involved with the p53 or the pRB/p16^INK4^. Other evidence, however, suggests that some genes are involved in immortalization but have nothing to do with the telomere status (Duncan et al., 2000). The target gene list of the overexpressed miRNAs (Supplementary Table S8) is associated with the MAPK signaling pathway (Supplementary Table S10). MAPK (mitogen-activated protein kinase) signaling pathway is a highly conserved module that is involved in various cellular functions, including cell differentiation, migration, and proliferation (Zhang and Liu, 2002; Mebratu and Tesfaigzi, 2009; Ables et al., 2011). The ErbB family of proteins contains 4 receptor tyrosine kinase receptors, which include EGFR/ERBB1/HER1, ERBB2/HER2, ERBB3/HER3, and EFBB4.HER4. Transphosphorylation of the ErbB dimer partners by activation of HER2 and EGFR stimulate intracellular pathways and STAT transcription factors. Of which, the EGFR and HER2 can activate STAT3 via phosphorylation through SRC to promote cell survival (Kavarthapu et al., 2021).

## CONCLUSIONS

In summary, a total of 422 miRNAs was identified in samples of the ADOL ZS-1 cell line and 188 of those miRNAs were differentially expressed between the ZS-1 and the primary chicken embryo fibroblast (CEF) samples. Bioinformatic analysis of the target gene list of the differentially expressed miRNAs that were only identified in the ZS-1 samples (not in the 21st passage CEFs) in contrast to the primary CEF samples suggested that those miRNAs might have modulated the cell immortalization processes and contributed to the maintenance of the immortalization state by targeting genes involved in various biological processes and key pathways. This study provided a piece of experimental and informatics evidence that the epigenetic factor, miRNAs, plays a role in modulating cell proliferation, senescence process and immortalization state.

## Appendix Supplementary materials

**Table S1.** A list of identified known and novel microRNAs from the primary and 21st passage chicken embryo fibroblasts and the ZS-1 cell line. Image, application 1

**Table S2.** MicroRNAs identified in the primary chicken embryo fibroblast samples. Image, application 2

**Table S3.** MicroRNAs identified in the 21st passage chicken embryo fibroblast samples. Image, application 3

**Table S4.** MicroRNAs identified in cells of the ZS-1 line. Image, application 4

**Table S5.** A list of miRNAs identified ONLY in one or two of the three types of cell samples. Image, application 5

**Table S6.** Lists of differentially expressed miRNAs of the ZS-1 cell & primary CEF contrast and the 21st passage CEF & the primary CEF contrast. Image, application 6

**Table S7.** Target genes of all differentially expressed miRNAs detected in ZS-1 cells in contrast to the primary CEFs. Image, application 7

**Table S8.** Target genes of the 18 upregulated miRNAs identified only in the ZS-1cells in contrast to the primary CEFs. Image, application 8

**Table S9.** Target genes of the 45 downregulated miRNAs identified only the ZS-1 cells in contrast to the primary CEFs. Image, application 9

**Table S10.** GO Terms and pathways associated with target genes of the upregulated miRNAs detected only in ZS-1 cells in contrast to the primary CEFs. Image, application 10

**Table S11.** GO Terms and pathways associated with target genes of the downregulated miRNAs detected only in ZS-1 cells in contrast to the primary CEFs. Image, application 11

## Footnotes

Supplementary material associated with this article can be found in the online version at doi:10.1016/j.psj.2022.102474.
